# 3D-printing-assisted flexible pressure sensor with a concentric circle pattern and high sensitivity for health monitoring

**DOI:** 10.1038/s41378-023-00509-z

**Published:** 2023-04-05

**Authors:** Jihun Lee, Hongyun So

**Affiliations:** 1 Department of Mechanical Engineering, Hanyang University, Seoul, 04763 South Korea; 2 Institute of Nano Science and Technology, Hanyang University, Seoul, 04763 South Korea

**Keywords:** Electrical and electronic engineering, Sensors

## Abstract

In this study, a flexible pressure sensor is fabricated using polydimethylsiloxane (PDMS) with a concentric circle pattern (CCP) obtained through a fused deposition modeling (FDM)-type three-dimensional (3D) printer and poly(3,4-ethylenedioxythiophene):poly(styrenesulfonate) (PEDOT:PSS) as the active layer. Through layer-by-layer additive manufacturing, the CCP surface is generated from a thin cone model with a rough surface by the FDM-type 3D printer. A novel compression method is employed to convert the cone shape into a planar microstructure above the glass transition temperature of a polylactic acid (PLA) filament. To endow the CCP surface with conductivity, PDMS is used to replicate the compressed PLA, and PEDOT:PSS is coated by drop-casting. The size of the CCP is controlled by changing the printing layer height (PLH), which is one of the 3D printing parameters. The sensitivity increases as the PLH increases, and the pressure sensor with a 0.16 mm PLH exhibits outstanding sensitivity (160 kPa^−1^), corresponding to a linear pressure range of 0–0.577 kPa with a good linearity of *R*^2^ = 0.978, compared to other PLHs. This pressure sensor exhibits stable and repeatable operation under various pressures and durability under 6.56 kPa for 4000 cycles. Finally, monitoring of various health signals such as those for the wrist pulse, swallowing, and pronunciation of words is demonstrated as an application. These results support the simple fabrication of a highly sensitive, flexible pressure sensor for human health monitoring.

## Introduction

Recently, flexible electronic devices that can sense important parameters (e.g., pressure, strain, and temperature) and/or wearable devices have been studied because of their various applications^1,2^, such as in health monitoring^3–6^, robotics^7–9^, and human‒machine interface sensing for virtual reality and/or sports monitoring^10–12^. Among these devices, flexible pressure sensors play a very important role in health monitoring by detecting blood pressure and heartbeat, which indicate human health conditions^13^. Based on their working mechanism, pressure sensors are generally categorized into four types: piezoresistive^14–18^, capacitive^19,20^, piezoelectric^21,22^, and triboelectric^23,24^. Among them, piezoresistive-type pressure sensors, which employ variations in the contact area between electrodes and detect resistance changes under applied pressure, have many advantages, such as high sensitivity, a simple device structure, and an easy read-out circuit^13,14^.

The parameters used to estimate the pressure sensor performance include the sensitivity, corresponding linear pressure range, response time, and durability. Among these parameters, a high sensitivity and a wide corresponding linear range are highly desired for utilizing the pressure sensor for practical applications^16^. In particular, the sensitivity is one of the most important factors. Because the working mechanism of piezoresistive-type pressure sensors is based on changes in the contact resistance when pressure is applied, the shape of the contact area (i.e., microstructure) is crucial to the sensitivity. Thus, to enhance the sensitivity, many researchers have investigated microstructures such as pyramids^2,25,26^, microdomes^18^, and micropillars^27^. In general, the polydimethylsiloxane (PDMS) elastomer is used to replicate microstructures and is a substrate of pressure sensors due to its flexibility and biocompatibility^2,18,27,28^. However, complex manufacturing processes, high costs, and a clean environment, including in lithography and etching processes, are required to fabricate these microstructures. Hence, simple, facile, low-cost processes that can eliminate the need for a clean room manufacturing environment are still required to create microstructures for high sensitivity of pressure sensors. To overcome the challenges in fabrication, many researchers have adopted three-dimensional (3D) printing technology, which is low cost, entails simple manipulations, and does not require a clean environment, as in lithography or etching processes, to create microstructures and fabricate flexible pressure sensors^29–31^. In particular, previous studies mimicked the human fingerprint to print a concentric circle pattern (CCP) by a direct ink writing (DIW) 3D printing method. The inks were composed of PDMS as a flexible substrate and incorporated conductive materials of carbon nanotubes (CNTs) and graphene. These sensors exhibited sensitivities of 2.08 and 2.4 kPa^−1^ in the low-pressure regions of 0.12 and 0.18 kPa, respectively^30,31^. However, the DIW 3D printing method, which prints one product at a time, has a disadvantage in scalable manufacturing processes compared to the casting method using molds. In addition, to effectively detect physiological signals of the human body, the sensitivity should be further enhanced, and the corresponding linear pressure range should be extended.

Among the various types of 3D printing technologies, fused deposition modeling (FDM)-type 3D printing technology has many advantages, such as a simple fabrication process, low material and device costs, a fast printing speed, and easily controllable fabrication parameters. However, this technology has a critical disadvantage in that products have rough surfaces due to the layer-by-layer printing. This leads to low quality of the product surface or poor properties (e.g., mechanical and thermal) with respect to the printing direction^32,33^. Although the FDM-type 3D printer includes defects, many researchers deliberately used its unique properties for useful applications, such as strain gauges^34^, anti-adhesion surfaces^35^, and micromixers^36^.

In this study, we demonstrated CCP microstructure-based flexible pressure sensors with PDMS and poly(3,4-ethylenedioxythiophene):poly(styrenesulfonate) (PEDOT:PSS) with high sensitivity and potential applications for human health monitoring. We proposed a novel design and manufacturing process for a CCP microstructure using an FDM-type 3D printer to utilize its specific properties (i.e., a rough surface). The initial CCP microstructure was composed of polylactic acid (PLA), a widely used filament in FDM-type 3D printers. In particular, the CCP-based microstructure was controlled by changing the printing layer height (PLH), which is one of the 3D printing parameters. To fabricate a flexible pressure sensor, we introduced casting processes to replicate the CCP surface (PLA plane) and cured flexible PDMS with the CCP surface. It was notable that PLA planes could be reused. PEDOT:PSS is a conductive polymer that is highly flexible, stretchable, and extremely cost-effective compared to other types of conducting media, such as Ag nanowires and CNTs^37^. Herein, the drop-casting method was employed to coat the PEDOT:PSS film on the cured PDMS. The CCP-based flexible pressure sensor exhibited a high sensitivity of 160 kPa^−1^, corresponding to a linear pressure range of 0–0.577 kPa. It also showed a stable and repeatable response under various pressures, durability under 4000 loading/unloading cycles, a response time of 114 ms, and a recovery time of 192 ms. Consequently, the developed pressure sensor was demonstrated to detect signals for human health monitoring, such as those for the wrist pulse during exercise and rest and for swallowing activity, and to distinguish the pronunciation of words in real time.

## Design and fabrication

### Design

**Fig. 1:**
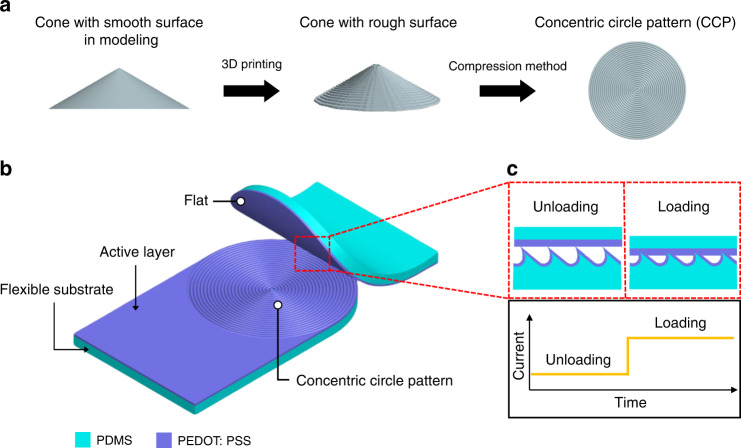
Conceptual illustration of a concentric circle pattern (CCP)-based flexible pressure sensor obtained through a 3D printer and its working mechanism. **a** Key idea of the fabrication of the CCP surface in-plane. **b** Configuration of the pressure sensor with a flat electrode and a CCP electrode, with each electrode including an active layer (PEDOT:PSS) and a flexible substrate (PDMS). **c** Working principle of the piezoresistive-type pressure sensor

Figure 1 illustrates the overall concept of a CCP-based flexible pressure sensor. We used an FDM-type 3D printer to create microstructures. As shown in Fig. 1a, the cone shape model has a smooth surface in computer-aided design (CAD). However, after 3D printing, the cone-shaped product has a rough surface because of the features of the FDM-type 3D printer. We deliberately utilized it to create a CCP microstructure. The top view shows that the printed cone model has concentric circles. To create a CCP surface in-plane, a method that converts surface structures from 3D to 2D is needed. In this study, we developed a novel compression method to convert a 3D structure into a planar microstructure. Figure 1b shows the CCP-based pressure sensor composed of CCP and flat electrodes. Each electrode consists of PDMS as a flexible substrate and PEDOT:PSS as an active layer. Figure 1c presents a working mechanism to sense current changes when pressure is applied. It involves a piezoresistive-type pressure sensor that detects resistance changes via geometrical deformation of the contact area. When pressure is applied, the contact area between the CCP and flat surfaces increases, inducing a decrease in the resistance. Thus, the current increases, and we record the current changes under external pressure. A cross-sectional schematic illustration of the pressure sensor in the unloaded and loaded states is presented in Fig. 1c. Because the initial contact area determines the pressure sensor sensitivity, the sizes of the microstructures in the CCP are important. Therefore, herein, we fabricate four different types of CCPs with respect to the PLH, which is one of the 3D printing parameters, to determine the resolution in the printing direction and compare the sensitivities obtained with each PLH type.

### Fabrication

**Fig. 2:**
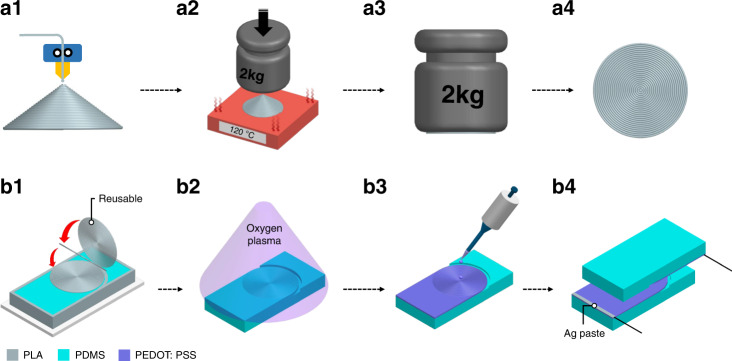
Schematic of the overall fabrication process, including the CCP surface in detail and pressure sensor based on the CCP with an active layer. **a1** Printing the PLA cone model using an FDM-type 3D printer; **a2** compressing the PLA with a weight above the glass transition temperature of PLA; **a3** cooling PLA in air; **a4** finalizing PLA with the CCP planar surface. **b1** Pouring PDMS onto the mold and covering the PLA plane with the CCP surface; **b2** peeling off the PLA plane and performing oxygen plasma treatment on the PDMS with the CCP surface; **b3** coating PEDOT:PSS by drop-casting for electrical conductivity; **b4** wiring for electrical connection, and assembling the CCP and flat surfaces for electrical contact

Figure 2 shows an overall schematic of the planar CCP and pressure sensor fabrication process. The process is divided into two parts: (1) fabrication of the CCP surface PLA plane and (2) fabrication of the pressure sensor using the PLA plane. Figure 2a shows the fabrication process for making a CCP surface PLA plane. First, we designed a cone model with an inner length, an inner height, an upper thickness, and an angle of 7 mm, 4.04 mm, 0.25 mm, and 30°, respectively, in CAD software (NX, Siemens) (see Fig. S1 in the Supplementary Information). Next, the cone model was printed by the FDM-type 3D printer (GUIDER IIs, FlashForge Co.), as shown in Fig. 2a1. The constant printing parameters, i.e., the printing speed, travel speed, extruder temperature, and platform temperature, were set to 60 mm/s, 80 mm/s, 220 °C, and 40 °C, respectively. Here, we only varied the PLH of the 3D printing parameter from 0.1 to 0.16 mm at 0.02 mm intervals. The 3D-printed cone model had a rough surface due to the specific property that the FMD-type 3D printer prints the model layer-by-layer, although a thin cone shape model was designed by the CAD system with a smooth surface, as shown in Fig. 1a. The 3D cone had to be deformed into the 2D plane while keeping its surface structure. To convert the cone structure from 3D to a 2D plane, we developed a novel compression method that heats the 3D cone structure to above the glass transition temperature of PLA and compresses the printed cone using a weight to form the pattern in the 2D plane. Figure 2a2 presents the compression process in which the products are heated on a hotplate at 120 °C for 10 s to make the products reach the glass transition temperature and the products are compressed. Here, the PLA was overheated compared with the glass transition temperature (55 − 70 °C) for fast heat transfer. As a result, the PLA deformed easily to convert into a 2D plane. Subsequently, we compressed the PLA cone with a 2-kg weight for 50 s to unfold the 3D structure into a 2D plane. A 2-kg weight was found to be the minimum weight for compression of all PLA cones with different PLHs (0.1, 0.12, 0.14, and 0.16 mm) to unfold them into a 2D plane. To demonstrate the uniformity of the unfolded 2D structures obtained using the compression method with a 2-kg weight for 50 s, a PLA cone with a 0.1 mm PLH in 3D was compressed (Fig. S2a, b in the Supplementary Information), and the thickness of the unfolded 2D structure was measured at three different positions using a digital vernier caliper (AOS Absolute Digimatic Caliper, Mitutoyo Co.), as shown in Fig. S2c. All of the measured thicknesses were the same at 0.31 mm, indicating that the 3D PLA cone structure was successfully unfolded into a 2D structure. The compression time on the hotplate during the compression method is the other condition determining the quality of the PLA plane. In this study, a compression time of 50 s was found to be the optimal condition to successfully create microstructures. For compression times >50 s (e.g., 2 and 5 min), the microstructures on the CCP surface were damaged or crumbled, as shown in Fig. S3. After the compression method, we cooled the structure to below the glass transition temperature of PLA to prevent deformation during compression while maintaining the CCP in the plane (Fig. 2a3). Finally, CCP surface PLA was successfully created in a 2D plane (Fig. 2a4), and corresponding optical images of PLA planes with respect to the PLH (0.1, 0.12, 0.14, and 0.16 mm) are presented in Fig. S4a–d in the Supplementary Information. It is noteworthy that the PLH enabled control of the concentric circle sizes (i.e., width, total perimeter, and depth). The novel compression method after printing PLA cones in 3D has two advantages compared to direct printing in 2D. First, direct printing in 2D using some 3D printers (e.g., a GUIDER IIs, FlashForge Co., which was used to print PLA cones in this study), cannot form CCP surfaces in 2D, as shown in Fig. S5a1 in the Supplementary Information. However, despite using the same printer, a CCP surface in 2D could be created by utilizing the compression method, as shown in Fig. S5a3. Second, although other FDM-type 3D printers (e.g., a 3DWOX 2X, Sindoh Co.) could directly print CCP surface in 2D (Fig. S5a2), the microstructures of the CCP surface in 2D were not sharply formed. Figure S5b1–3 show the top surface views of PDMS replicated from PLA fabricated by direct printing with a GUIDER IIs, direct printing with a 3DWOX 2X, and the compression method with a GUIDER IIs, respectively. Although the top view of the replicated PDMS obtained through direct printing with the 3DWOX 2X showed the CCP (Fig. S5b2), CCP microstructures were only formed for the replicated PDMS fabricated using the compression method, as shown in Fig. S5c1–3.

Figure 2b shows the fabrication process of the pressure sensor using the created CCP surface PLA plane in Fig. 2a. The prepared PDMS (Sylgard 184, Dow Corning Inc.), which was mixed in a 10:1 ratio (prepolymer:curing agent) and degassed in a vacuum chamber for 1 h, was poured onto the mold and sealed the PLA plane (Fig. 2b1). The PDMS was then baked on a heater at 50 °C for 12 h for perfect curing. Because PLA returns to its original shape (i.e., cone) above the glass transition temperature, it must be baked below the glass transition temperature of PLA. The thickness and width of the cured PDMS were 4 and 15 mm after the baking process, respectively. After the curing process, the PLA plane was peeled off, and it was noteworthy that the PLA plane could be reused. Next, cured PDMS with the CCP surface was treated with oxygen plasma treatment (CUTE-1MPR, Femto Science Inc.) for 2 min to improve the hydrophilicity of the PDMS and evenly disperse the PEDOT:PSS solution (1.1% in H_2_O, Sigma‒Aldrich) on the PDMS (Fig. 2b2)^38^. It is difficult to wet a PEDOT:PSS solution on PDMS because it has a low surface energy. However, oxygen plasma treatment allows a PEDOT:PSS solution to wet PDMS due to the increase in surface energy induced by the creation of hydrophilic –OH functional groups on the PDMS surface^39,40^. We employed PEDOT:PSS as a conductive material because it is flexible, stretchable, and low cost compared to the generally used conductive materials of Ag nanowires and CNTs^37^. There are many methods to coat PEDOT:PSS, such as drop-casting^26,41,42^, dip-coating^43^, oxidative chemical vapor deposition^44^, spin-coating^45^, and spraying^46^. To endow PDMS with conductivity, we employed the drop-casting method because it has many advantages, such as being low-cost, easy, and quick, and it can uniformly coat microstructures with peaks^26,41,42^. After the oxygen plasma treatment, the PEDOT:PSS solution was dropped on the PDMS surface using four droplets (80 µL for each droplet) to cover the whole surface of the PDMS, which became an active layer. Subsequently, the substrates were heated on a hotplate at 100 °C for 1 h to dry the water in the PEDOT:PSS solution. As a result, PEDOT:PSS was coated on the CCP surface, as shown in Fig. 2b3. Next, wires with Ag paste for electrical contact were connected. After that, the CCP and flat surface electrodes were assembled. Finally, various CCP-based flexible pressure sensors with respect to the PLH were created (Fig. 2b4).

## Results and discussion

**Fig. 3:**
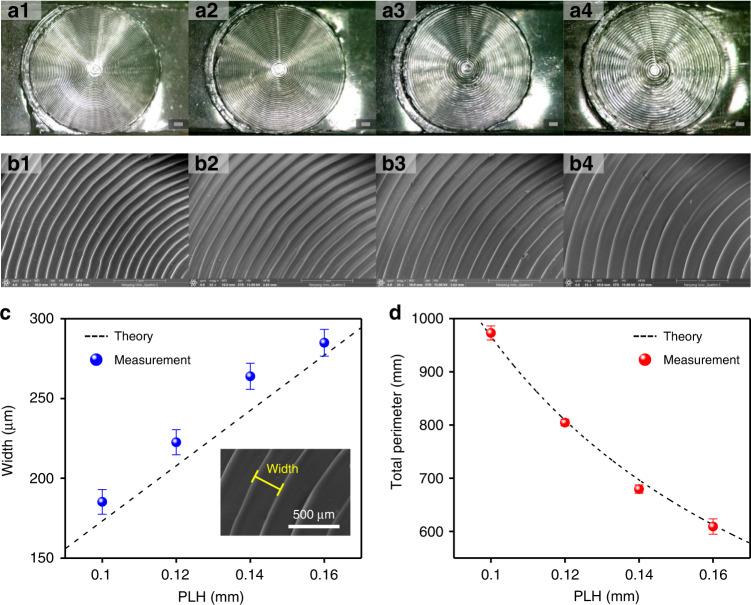
Optical and SEM images of cured CCP surface PDMS with respect to the PLH, and theoretical and measured values of the width and total perimeter of CCP structures. Optical images of PDMS based on CCP surfaces with four different PLHs: **a1** 0.1 mm; **a2** 0.12 mm; **a3** 0.14 mm; and **a4** 0.16 mm. SEM images of each CCP surface: **b1** 0.1 mm; **b2** 0.12 mm; **b3** 0.14 mm; and **b4** 0.16 mm. (Scale bar = 1 mm.) Theoretical and measured values of the **c** width (inset: 0.16 mm PLH) and **d** total perimeter of the CCP microstructures with respect to the PLH

Figure 3 shows the optical and scanning electron microscopy (SEM) images of each created PDMS with the CCP with respect to the PLH and sizes (i.e., width and total perimeter) of the CCP structures and a comparison of theoretical calculation and experimental measurement results. To take optical and SEM images, the CCP surface PDMS before oxygen plasma treatment in Fig. 2b2 was employed. Figure 3a1–4 present optical images of each cured PDMS with respect to the PLH (0.1, 0.12, 0.14, and 0.16 mm). Figure 3b1–4 show the SEM images of CCP surfaces with respect to the PLH (0.1, 0.12, 0.14, and 0.16 mm) in detail. The width and distance between tips were uniform for each PLH, and the width increased as the PLH increased. Interestingly, the width with respect to the PLH was calculated by theory. Figure S6a, b show the theory of the width calculation. The width of each CCP can be expressed by

1 $${{{\mathrm{w}}}} = \sqrt 3 h$$

where *w* and *h* are the width and PLH in mm units, respectively. A more detailed derivation procedure for Eq. 1 is explained in Fig. S6 in the Supplementary Information. According to Eq. 1, the theoretical values of the width were 173, 208, 242, and 277 µm for 0.1, 0.12, 0.14, and 0.16 mm PLHs, respectively. In addition, to compare theoretical and experimental values, we measured the width of the CCP with respect to the PLH based on the SEM images (see the inset SEM image for the 0.16 mm PLH in Fig. 3c). For measurements, the ImageJ (National Institutes of Health) program was utilized to measure the width based on the SEM images (Fig. 3b). The measured widths were 185, 222, 264, and 285 µm for 0.1, 0.12, 0.14, and 0.16 mm PLHs, respectively, as shown in Fig. 3c. These results show that the width measurements exhibited good linearity, with a 0.984 coefficient of determination (*R*^2^), and were similar to theoretical values. It is noteworthy that all measured values were higher than the theoretical values. One possible reason is that the PLA planes were slightly stretched in the plane direction when they were pushed (collapsed) through the compression method (Fig. 2a2). We also analyzed the total perimeter of the CCPs using the width values, and it was also obtained theoretically. Figure S6c in the Supplementary Information illustrates how to calculate the total perimeter in detail. The total perimeter of each CCP can be expressed by

2 $$P_{{{{\mathrm{total}}}}}{{{ = 22.66}}}\left(\frac{{{{{4.165}}}}}{h}{{{ + 1}}}\right)$$

where P_total_ and h are the total perimeter and PLH, respectively. The theoretical values of the total perimeter were 966.4, 809.2, 696.8, and 612.5 mm for 0.1, 0.12, 0.14, and 0.16 mm PLHs, respectively. To estimate the total perimeter values, optical images of CCPs on PDMS were employed. ImageJ was also utilized to measure the total perimeter based on the optical images. Consequently, the measured total perimeters were 973.2, 804.5, 679.5, and 609.1 mm for 0.1, 0.12, 0.14, and 0.16 mm PLHs, respectively, as shown in Fig. 3d. These results showed that the total perimeter decreased as the PLH increased; in contrast, the width of the CCP increased. The measured values followed their theoretical values well according to both the width and total perimeter results. These results explained why the tendencies with respect to the PLH occurred. In addition, the depth of the microstructures with respect to the PLH was measured using the ImageJ program and cross-sectional SEM images of the CCP surface, as shown in Fig. S7a–d in the Supplementary Information. The depth was measured to be 78.2, 83.9, 102, and 129 µm for 0.1, 0.12, 0.14, and 0.16 mm PLHs, respectively, as shown in Fig. S7e. It is noteworthy that the depth of the microstructures increased as the PLH increased, which is the same tendency as that between the width of the microstructures and PLH.

**Fig. 4:**
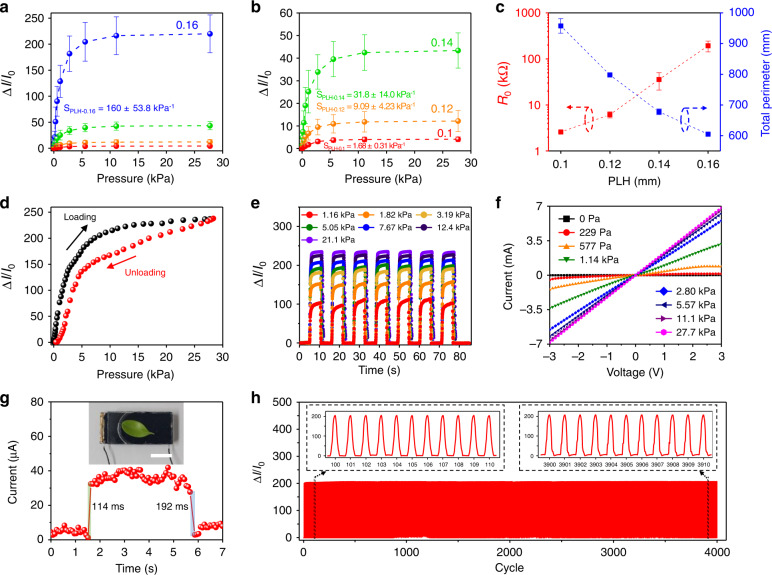
Characterization of the sensing performance of the CCP-based pressure sensor. **a** Sensitivity of the 0.16-mm PLH pressure sensor. **b** Magnified graph in **a** and sensitivities of 0.1-mm, 0.12-mm, and 0.14-mm PLHs pressure sensors, excluding the 0.16-mm PLH sensor. **c** Comparison of initial contact resistances and total perimeters with respect to the PLH. **d** Hysteresis for a loading and unloading cycle with a maximum pressure of 28.3 kPa. **e** Dynamic response under seven different pressures repeated for seven cycles. **f** Current-voltage (*I–V*) curves for different static pressures. **g** Response and recovery times with an applied leaf load of 26 mg (scale bar = 10 mm). **h** Durability test for 4000 cycles under a 6.56 kPa pressure. The insets present magnified plots of the 100 to 110 and 3900 to 3910 cycles. A CCP-based pressure sensor with a 0.16 mm PLH was used in **d**, **e**, **f**, **g**, and **h**

After the entire pressure sensor fabrication, we evaluated the performance of the pressure sensor, which comprised the CCP and a flat surface with a PEDOT:PSS conductive layer coated on PDMS, with respect to the PLH. To evaluate the performance of the pressure sensor, the current between the CCPs and flat surface electrodes was measured by applying a constant voltage of 1 V using a source meter (SMU 2611B, Keithley Instruments Inc.). Among the sensing performance parameters, the sensitivity is a critical parameter for pressure sensing. To characterize the sensitivity of the pressure sensor, current changes were measured by applying a static pressure through a weight on the sensing area, which was the CCP region with a 15-mm diameter. The weights were 0.5, 1.5, 2.5, 5.4, 10.4, 20.5, 50.4, 100.4, 200.3, and 500.2 g, and the corresponding pressures produced were 28, 83, 139, 299, 577, 1137, 2795, 5568, 11108, and 27739 Pa, respectively^47^. As the different pressures were applied, the currents were measured for 20 s. Meanwhile, the current responses were expressed as the current change (Δ*I*) from the initial current (*I*_0_) divided by the initial current (*I*_0_), that is, Δ*I*/*I*_0_. Figure 4a, b show the current response of the pressure sensor with respect to the PLH under an applied pressure ranging from 0 to 27.7 kPa. The number of sensors used to obtain a current response was three for each PLH. The error bars for each PLH in Fig. 4a, b indicate the sensor-to-sensor uniformity. To quantitatively analyze the uniformity, a coefficient of variation (CV = standard deviation/average × 100%) was introduced. For the CCP-based pressure sensor with a 0.16 mm PLH, the CV was ~16.8% at 11.1 kPa. These sensors exhibited high uniformity compared to the pressure sensor with a random porous structure in a previous report (CV of 69.65% at a pressure of 10 kPa)^48^. The current responses of the pressure sensors rapidly increased in the low-pressure range (0–2.8 kPa) but saturated in the high-pressure range (>2.8 kPa) for all PLH pressure sensors. The sensitivity (*S*) of the pressure sensor is defined as δ(Δ*I*/*I*_0_)/δ*P*, where *P* is the applied pressure (kPa). In this study, to compare the sensitivities to pressure on the CCPs, we set the same pressure range (0–0.577 kPa) and calculated the sensitivities of each pressure sensor. The pressure sensor with a 0.16 mm PLH presented a high sensitivity of 160 ± 53.8 kPa^−1^, as shown in Fig. 4a. The 0.1, 0.12, and 0.14 mm PLH pressure sensors had sensitivities of 1.68 ± 0.31, 9.09 ± 4.23, and 31.8 ± 14.0 kPa^−1^, respectively, as shown in Fig. 4b. Accordingly, the CCP-based pressure sensor with a 0.16 mm PLH exhibited outstanding sensitivity that was 95.2 times higher than that with a 0.1 mm PLH. Therefore, the CCP-based pressure sensor with a 0.16 mm PLH benefits in detecting external pressure. Notably, the sensitivities increased from 1.68 to 160 kPa^−1^ with increasing PLH from 0.1 to 0.16 mm. This tendency can be attributed to the initial contact area. Because the sensitivity and working range of sensors differ depending on the PLH, a suitable sensor can be selected for particular applications (e.g., a high-sensitivity sensor (0.16 mm PLH) for gas flows and acoustic sound waves and a wide-working-range sensor (0.1 mm PLH) for intrabody pressure monitoring). Figure 4c exhibits the relationship between the initial resistance and total perimeter with respect to the PLH. The total perimeter has an indirect relation with the initial resistance instead of the contact area, but it is sufficient to explain the initial resistance. The average initial resistances were measured as 2.58, 6.15, 35.7, and 193 kΩ for 0.1, 0.12, 0.14, and 0.16 mm PLHs, respectively. A high initial resistance means that the initial current is very low because the same voltage of 1 V is applied. To improve the sensitivity, the initial current (*I*_0_) should be reduced because the available variation range increases when the initial current decreases^16,49,50^. A small initial contact area between the electrodes causes the initial current to decrease. Therefore, to enhance the pressure sensor sensitivity, the microstructure contact area must be small. The total perimeter under the initial resistance was measured to be 957.5, 797.8, 677.7, and 604.7 mm for 0.1, 0.12, 0.14, and 0.16 mm PLHs, respectively. Because the total perimeter and initial contact area are proportionally related, a short total perimeter means a small initial contact area, which induces a reduction in the initial current. Hence, the initial current decreases as the total perimeter decreases. From the total perimeter theory and measurements in Fig. 3d, the total perimeter decreased when the PLH increased. In summary, increasing the PLH decreases the total perimeter and reduces the initial contact area. In addition, a reduced initial contact area induces an increase in the initial resistance. Because an increased initial resistance means that the initial current is reduced, the sensitivity is improved. Consequently, the sensitivity increases with increasing PLH. In addition, the increased depth of microstructures as the PLH increased could create a comparable contact area under applied pressure, which generated the similar maximum current values between the pressure sensors with 0.1 and 0.16 mm PLHs (i.e., 2.31 and 2.36 mA under 27.7 kPa for 0.1 and 0.16 mm PLHs). Thus, the pressure sensor with a 0.16 mm PLH, which exhibited the lowest initial current (*I*_0_), showed the highest sensitivity (δ(Δ*I*/*I*_0_)/δP). In other words, the depth of the microstructure contributed to enhancing the sensitivity as the PLH increased. These results reveal that the sensitivity of the pressure sensor increased as the PLH increased. However, above the 0.16 mm PLH (i.e., 0.18 and 0.2 mm PLHs), the cone model could not be printed by a 3D printer under the same conditions because the supporting area between each layer decreased, and it was not sufficient to support the layer.

In this study, we employed the CCP-based pressure sensor with a 0.16 mm PLH, which exhibited the highest sensitivity of 160 kPa^−1^ and a good linearity with an average *R*^2^ of 0.978 in the linear range of 0–0.577 kPa, to characterize other performance parameters of the pressure sensor, such as the hysteresis, repeatability, *I–V* curve, response and recovery times, and durability. To characterize the hysteresis and dynamic response for repeatability, a tensile and compression testing machine (MCT-2150, A&D Co.) was used with a loading and unloading speed of 30 mm/min. The hysteresis of the pressure sensor was investigated by loading and unloading the pressure. Figure 4d presents the hysteresis of the pressure sensor for a loading and unloading pressure of 28.3 kPa for one cycle. The current response curves corresponding to the loading and unloading of pressure formed a hysteresis loop. To quantitatively analyze the hysteresis of the pressure sensor, the degree of hysteresis (DH) was evaluated as the ratio of the area difference between loading and unloading (Δ*A* = *A*_*loading*_ − *A*_*unloading*_) divided by the area of the loading curve (*A*_*loading*_), that is, DH = Δ*A*/*A*_*loading*_ ×100%. Accordingly, the piezoresistive-type CCP-based pressure sensor with a 0.16 mm PLH exhibited a DH of 16.57%. Hysteresis is a common issue in flexible piezoresistive-type pressure sensors due to mainly the viscoelastic properties of the elastomer (e.g., PDMS) and weak interactions between the conductive material and the elastomer;^48,51^ however, the tradeoff is a high sensitivity resulting from the large deformation of the elastomer^52^. In this study, the CCP-based pressure sensor with a 0.16 mm PLH showed an outstanding sensitivity of 160 kPa^−1^. Figure 4e shows the dynamic responses and the current response when pressure is loaded onto and unloaded from the pressure sensor for seven different external pressures from 1.16 to 21.1 kPa for seven cycles. The results showed that the current responses were stable and repeatable under various and repeated pressures. Furthermore, as the pressure increased, the increase rate of the response gradually decreased because the pressure sensor saturated over the pressure of 2.8 kPa. Figure 4f shows the pressure sensor current-voltage (*I–V*) curves under static pressures from 0 to 27.7 kPa. The voltages were swept from −3 to 3 V during static pressure application. The *I–V* curve results presented linear relations between the currents and voltages. These results indicated that the pressure sensor exhibited ohmic contact behavior. To evaluate the response and recovery times, the current was measured while a tiny leaf of 26 mg was on the pressure sensor, as shown in Fig. 4g. This result indicated that our device could detect a tiny weight as small as a leaf. When the small load was applied, the pressure sensor exhibited response and recovery times of 114 and 192 ms, respectively. To evaluate the durability of the pressure sensor, the current responses were measured for 4000 cycles of loading and unloading under 6.56 kPa using the tensile and compression testing machine. As shown in Fig. 4h, there was no significant deterioration of the current responses. In addition, the beginning and end of the current responses from the 100 to 110 and from the 3900 to 3910 cycles, respectively, showed the stability, steadiness, and repeatability of the output signals (see inset graphs in Fig. 4h). Therefore, these results supported that the pressure sensor is durable.

Table 1 compares the pressure sensors between this study and previous research on flexible pressure sensor performance, including in terms of the manufacturing methods based on 3D printing, materials, sensitivity, corresponding linear pressure range, and response and recovery times^29–31,53–58^. All references were published after 2019. Herein, we list research based on 3D printing manufacturing of pressure sensors. By comparing the performance, we presented the situations in which our device has a benefit. Although our device response and recovery times were slow compared to those in other research^29–31,53,57^, our pressure sensor showed an outstanding sensitivity of 160 kPa^−1^ in a low-pressure range (0–0.577 kPa). In particular, the developed pressure sensor with the CCP was more sensitive in the low-pressure range and had a wide linear pressure range compared with previous studies that fabricated microstructures of concentric circles for utilization in pressure sensors^30,31^. These results support that the proposed pressure sensor has potential applications in sensitively detecting subtle external signals, such as the wrist pulse for humans.

**Table 1:** Comparison table of flexible pressure sensors based on 3D printing fabrication

3D printing method	Materials (substrate/conductive layer)	Sensitivity (kPa^−1^)	Corresponding linear range (kPa)	Response time (ms)	Recovery time (ms)	Ref
DIW	PDMS/CB, MWCNT, Cu	255	50–300	32	61	^29^
DIW	PDMS/CNT	2.08	<0.12	50	N/A	^30^
DIW	PDMS/Graphene	2.4	<0.18	60	N/A	^31^
DIW	TPU/CB	5.54	<10	~20	~30	^53^
DIW	Ecoflex/CNT, SiNP	0.096	0–175	N/A	N/A	^54^
DLP	DN ionic conductive hydrogel	0.06	0–5	320	400	^55^
DLP	Conductive PAAm-PEGDA hydrogel	0.91	0–2	~200	~500	^56^
DLP	TPU/CNT	1.02	0–130	65	29	^57^
DLP	PU/CNT	0.111	0–10	N/A	N/A	^58^
FDM	PDMS/PEDOT:PSS	160	0–0.577	114	192	This work

*DIW* Direct ink writing, *DLP* Digital light processing, *FDM* Fused deposition modeling, *PDMS* Polydimethylsiloxane, *CB* Carbon black, *MWCNT* Multiwalled carbon nanotube, *CNT* Carbon nanotube, *TPU* Thermoplastic polyurethane, *SiNP* Silica nanoparticle, *DN* Double-network, *PAAm-PEGDA* Polyacrylamide-polyethylene glycol diacrylate, *PU* Polyurethane, *PEDOT:PSS* Poly(3,4-ethylenedioxythiophene):poly(styrenesulfonate)

### Applications

**Fig. 5:**
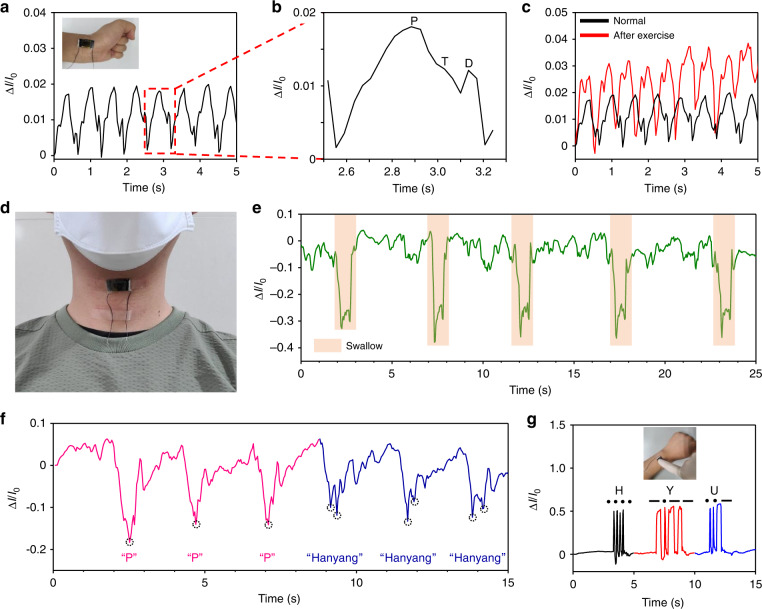
Applications of the CCP-based pressure sensor with a 0.16 mm PLH for human health monitoring. **a** Radial artery pulses on the wrist when in a normal state (inset: pressure sensor attached to the wrist). **b** Zoomed-in view of one periodic pulse in a presenting the P, T, and D waves. **c** After exercising and compared with the normal state. **d** Pressure sensor attached on the neck. Responses to **e** swallowing activity and **f** pronunciation of “P” and “Hanyang” in real time. **g** Reading Morse codes for H, Y, and U, produced by touching the pressure sensor attached to the skin

To investigate the possible usages of the pressure sensor for monitoring human physiological signals such as artery pulse waves, swallowing, and pronunciation, a fabricated CCP-based pressure sensor with a 0.16 mm PLH was attached to the wrist and neck. The fabrication process of the pressure sensor for detecting human physiological signals is shown in Fig. S8 in the Supplementary Information, which was based on the previous manufacturing process (Fig. 2b) using the reusable CCP PLA plane with a 0.16 mm PLH. The thickness of a flexible pressure sensor is one of the important parameters affecting the detection of subtle pressure for human health monitoring (e.g., wrist pulse) and the comfort of wearable devices under stretching or bending. To investigate the thickness effect on the device performance, various electrode thicknesses (i.e., 0.4, 1, and 2 mm) were characterized for wrist pulse measurement, as shown in Fig. S9a–c in the Supplementary Information. To measure the wrist pulse in the electrical signal, pressure sensors with different thicknesses were placed at the same position, and current responses were recorded for 5 s. The current response of each pressure sensor tended to increase as the thickness decreased, as shown in Fig. S9d. These results imply that a thin pressure sensor is more effective for measuring subtle pressure than thicker devices. In addition, as the thickness decreases, the pressure sensor becomes light and easier to bend. Hence, we selected the lowest thickness of 0.4 mm, which showed the highest current responses compared to the other thicknesses, for utilization in health monitoring. Figure 5a–c show the real-time signals of a wrist pulse, which correspond to the pulse of the radial artery in the wrist in a normal state and after exercise. The device was attached to the wrist, and the current responses were measured (see inset in Fig. 5a). The sensor was able to detect subtle periodic signals as small as a wrist pulse, as shown in Fig. 5a. The percussion wave (P), tidal wave (T), and diastolic wave (D), which are typical waves shown in normal wrist pulses, were detected by zooming-in on one periodic pulse, as shown in Fig. 5b. The corresponding heart rate was ~93 beats per minute in the normal state. Figure 5c presents wrist pulses for the normal state and after exercise. After exercise, the heartbeat is increased by ~110 beats per minute and is amplified compared to the normal state. This finding supports that our pressure sensor can be employed to monitor wrist pulses for physical health training or monitoring. To demonstrate the performance of the sensor on a curved surface, the sensor was additionally attached to the curved surface of the wrist by rotating it 90°, as shown in Fig. S10a, b in the Supplementary Information. Figure S10c shows a comparison of the current response for the wrist pulse on flat and curved surfaces. The maximum current responses of the device on flat and curved surfaces were recorded as 0.018 and 0.011, respectively. These results showed that the current response decreased when the flexible pressure sensor was bent. This might be because the increased normal force resulting from the bending motion increased the initial contact area between the microstructures and flat electrodes, which also induced an increase in the initial current (*I*_0_) such that the overall current response decreased (Δ*I*/*I*_0_). Although the sensitivity slightly decreased, the sensor still exhibited a stable response on the curved surface. To further demonstrate potential applications for health monitoring, the CCP-based pressure sensor was attached to the neck and used to estimate the signals of swallowing and pronunciation. Figure 5d shows the device attached to the neck. As shown in Fig. 5e, when water was repeatedly swallowed five times, the current signal could recognize the swallowing activity in real time. Thus, our device can be used for people who have difficulty eating or drinking by monitoring whether they swallow or not. Moreover, the CCP-based pressure sensor distinguished the pronunciation of two utterances, “P” and “Hanyang,” which were monosyllabic and disyllabic, respectively, as shown in Fig. 5f. When the speaker pronounced “P” three times, the corresponding current response was similar each time and exhibited one peak. Subsequently, after pronouncing “P,” the speaker said “Hanyang” three times, and the corresponding shapes presented two peaks. Thus, these results showed that the pressure sensor distinguished the pronunciation based on a comparison of the current shapes. Figure 5g shows the device attached to the back of a hand (see the inset in Fig. 5g) and the corresponding current responses upon touching the Morse code of H, Y, and U. The dash and dot of Morse code were represented by the long and short current responses generated by a long touch and a short touch, respectively. These results showed that the device recognized even Morse code (e.g., H, Y, and U) on the skin by touching, supporting the use of the fabricated sensor as a haptic device.

## Conclusion

In conclusion, herein, we successfully developed a CCP in a 2D plane using an FDM-type 3D printer and utilized it to fabricate a flexible piezoresistive-type pressure sensor with a PEDOT:PSS conductive layer. To create a CCP surface on the PDMS substrate with conductivity, the drop-casting method was employed to coat PEDOT:PSS on the CCP surface. The CCPs were fabricated into four types by changing the PLH from 0.1 to 0.16 mm at intervals of 0.02 mm. The sizes of the CCPs (i.e., width, total perimeter, and depth) were controlled by changing the PLH and could be theoretically calculated. The theoretical values of the width and total perimeter with respect to the PLH well matched the experimental results. After fabrication, the CCP-based pressure sensors were characterized by measuring the current under applied pressures. After fabrication of the CCP-based pressure sensors, the sensitivity with respect to the PLH was estimated. The results showed that the sensitivity was enhanced with increasing PLH. As a result, the CCP-based pressure sensor with a 0.16 mm PLH exhibited an outstanding sensitivity of 160 kPa^−1^ with a linear pressure range from 0 to 0.577 kPa (*R*^2^ = 0.978). Because the initial current decreased as the initial contact area decreased, the sensitivity improved when the total perimeter decreased. From the theoretical and experimental results of the total perimeter of the CCP, the total perimeter decreased when the PLH increased, which induced a decrease in the initial current. Thus, this explained why the sensitivity increased as the PLH increased. Furthermore, we conducted further characterization of the performance using the CCP-based pressure sensor with a 0.16 mm PLH. The device showed an output with good repeatability and stability, including excellent stability over 4000 cycles under a 6.56 kPa pressure. Because the pressure sensor was flexible, attachable to human skin, and exhibited high sensitivity at a small pressure range, it was suitable for healthcare monitoring. The pressure sensor was attached to the wrist and demonstrated the ability to collect radial artery pulses in normal and exercise states in real time. It could also be attached to the neck and recognize swallowing activity and the pronunciation of monosyllabic and two-syllabic words in real time. Additionally, the device on the skin recorded the Morse code of H, Y, and U as responses to long and short touches. In summary, the findings of this study support applications in health monitoring and wearable medical devices of the high-performance CCP-based flexible pressure sensors obtained via simple and cost-effective 3D printing and compression manufacturing methods.

## Supplementary information

Supplementary Information

## References

[1] Yang JC (2019). Electronic skin: Recent progress and future prospects for skin‐attachable devices for health monitoring, robotics, and prosthetics. Adv. Mater..

[2] Bae GY (2018). Pressure/temperature sensing bimodal electronic skin with stimulus discriminability and linear sensitivity. Adv. Mater..

[3] Liu Y (2017). Lab-on-skin: A review of flexible and stretchable electronics for wearable health monitoring. ACS Nano.

[4] Fu X, Dong H, Zhen Y, Hu W (2015). Solution-processed large-area nanocrystal arrays of metal-organic frameworks as wearable, ultrasensitive, electronic skin for health monitoring. Small..

[5] Pang Y (2018). Epidermis microstructure inspired graphene pressure sensor with random distributed spinosum for high sensitivity and large linearity. ACS Nano.

[6] Guo Y, Zhong M, Fang Z, Wan P, Yu G (2019). A wearable transient pressure sensor made with MXene nanosheets for sensitive broad-range human–machine interfacing. Nano Lett..

[7] Mousavi S, Howard D, Zhang F, Leng J, Wang CH (2020). Direct 3D printing of highly anisotropic, flexible, constriction-resistive sensors for multidirectional proprioception in soft robots. ACS Appl. Mater. Interfaces..

[8] Yan J (2020). Flexible and high-sensitivity piezoresistive sensor based on MXene composite with wrinkle structure. Ceram. Int..

[9] Pei Z (2021). A fully 3D‐printed wearable piezoresistive strain and tactile sensing array for robot hand. Adv. Mater. Technol..

[10] Sun Z, Zhu M, Shan X, Lee C (2022). Augmented tactile-perception and haptic-feedback rings as human-machine interfaces aiming for immersive interactions. Nat. Commun..

[11] Bai Z (2022). Constructing highly tribopositive elastic yarn through interfacial design and assembly for efficient energy harvesting and human-interactive sensing. Nano Energy.

[12] Gao S (2021). A motion capturing and energy harvesting hybridized lower‐limb system for rehabilitation and sports applications. Adv. Sci..

[13] Zang Y, Zhang F, Di C, Zhu D (2015). Advances of flexible pressure sensors toward artificial intelligence and health care applications. Mater. Horizons..

[14] Li S, Li R, González OG, Chen T, Xiao X (2021). Highly sensitive and flexible piezoresistive sensor based on c-MWCNTs decorated TPU electrospun fibrous network for human motion detection. Compos. Sci. Technol..

[15] Zhao T (2019). Highly sensitive flexible piezoresistive pressure sensor developed using biomimetically textured porous materials. ACS Appl. Mater. Interfaces..

[16] Shi J (2018). Multiscale hierarchical design of a flexible Piezoresistive pressure sensor with high sensitivity and wide linearity range. Small..

[17] Luo C (2017). A new approach for ultrahigh-performance piezoresistive sensor based on wrinkled PPy film with electrospun PVA nanowires as spacer. Nano Energy.

[18] Zhang Y (2017). Flexible and highly sensitive pressure sensor based on microdome-patterned PDMS forming with assistance of colloid self-assembly and replica technique for wearable electronics. ACS Appl. Mater. Interfaces..

[19] Jung Y (2021). Linearly sensitive pressure sensor based on a porous multistacked composite structure with controlled mechanical and electrical properties. ACS Appl. Mater. Interfaces..

[20] Chen S (2021). Multi-sized planar capacitive pressure sensor with ultra-high sensitivity. Nano Energy.

[21] Yang Y (2020). Flexible piezoelectric pressure sensor based on polydopamine-modified BaTiO3/PVDF composite film for human motion monitoring. Sens. Actuators A Phys.

[22] Wu Y, Ma Y, Zheng H, Ramakrishna S (2021). Piezoelectric materials for flexible and wearable electronics: a review. Mater. Des..

[23] Fan F-R (2012). Transparent triboelectric nanogenerators and self-powered pressure sensors based on micropatterned plastic films. Nano Lett.

[24] Garcia C, Trendafilova I, Guzman de Villoria R, Sanchez del Rio J (2018). Self-powered pressure sensor based on the triboelectric effect and its analysis using dynamic mechanical analysis. Nano Energy.

[25] Li H (2018). Ultrahigh-sensitivity piezoresistive pressure sensors for detection of tiny pressure. ACS Appl. Mater. Interfaces..

[26] Choong C-L (2014). Highly stretchable resistive pressure sensors using a conductive elastomeric composite on a micropyramid array. Adv. Mater..

[27] Park H (2015). Stretchable array of highly sensitive pressure sensors consisting of polyaniline nanofibers and Au-coated polydimethylsiloxane micropillars. ACS Nano.

[28] Mannsfeld SCB (2010). Highly sensitive flexible pressure sensors with microstructured rubber dielectric layers. Nat. Mater..

[29] Li T (2022). 3D printing of a flexible inclined‐tip cone array‐based pressure sensor. Adv. Mater. Technol..

[30] Wang H (2019). 3D‐printed flexible tactile sensor mimicking the texture and sensitivity of human skin. Adv. Mater. Technol..

[31] Wang H, Cen Y, Zeng X (2021). Highly sensitive flexible tactile sensor mimicking the microstructure perception behavior of human skin. ACS Appl. Mater. Interfaces..

[32] Torrado AR (2015). Characterizing the effect of additives to ABS on the mechanical property anisotropy of specimens fabricated by material extrusion 3D printing. Addit. Manuf..

[33] Prajapati H, Ravoori D, Woods RL, Jain A (2018). Measurement of anisotropic thermal conductivity and inter-layer thermal contact resistance in polymer fused deposition modeling (FDM). Addit. Manuf..

[34] Shin S, Ko B, So H (2022). Structural effects of 3D printing resolution on the gauge factor of microcrack-based strain gauges for health care monitoring. Microsystems Nanoeng.

[35] Sung J, So H (2021). 3D printing-assisted fabrication of microgrid patterns for flexible antiadhesive polymer surfaces. Surfaces and Interfaces.

[36] Koo D, So H (2022). Facile microfabrication of three dimensional-patterned micromixers using additive manufacturing technology. Sci. Rep..

[37] Fan X (2019). PEDOT:PSS for flexible and stretchable electronics: modifications, strategies, and applications. Adv. Sci..

[38] Liu H (2021). Harnessing the wide-range strain sensitivity of bilayered PEDOT:PSS films for wearable health monitoring. Matter.

[39] Lipomi DJ, Bao Z (2011). Stretchable, elastic materials and devices for solar energy conversion. Energy Environ. Sci..

[40] Juárez-Moreno JA, Ávila-Ortega A, Oliva AI, Avilés F, Cauich-Rodríguez JV (2015). Effect of wettability and surface roughness on the adhesion properties of collagen on PDMS films treated by capacitively coupled oxygen plasma. Appl. Surf. Sci..

[41] Valero A, Mery A, Gaboriau D, Gentile P, Sadki S (2019). One step deposition of PEDOT–PSS on ALD protected silicon nanowires: toward ultrarobust aqueous microsupercapacitors. ACS Appl. Energy Mater.

[42] Manjakkal L, Pullanchiyodan A, Yogeswaran N, Hosseini ES, Dahiya R (2020). A wearable supercapacitor based on conductive PEDOT:PSS‐coated cloth and a sweat electrolyte. Adv. Mater..

[43] Ding Y, Yang J, Tolle CR, Zhu Z (2018). Flexible and compressible PEDOT:PSS@Melamine conductive sponge prepared via one-step dip coating as piezoresistive pressure sensor for human motion detection. ACS Appl. Mater. Interfaces..

[44] Clevenger M, Kim H, Song HW, No K, Lee S (2021). Binder-free printed PEDOT wearable sensors on everyday fabrics using oxidative chemical vapor deposition. Sci. Adv..

[45] Andrei V (2017). Size dependence of electrical conductivity and thermoelectric enhancements in spin-coated PEDOT:PSS single and multiple layers. Adv. Electron. Mater..

[46] Bae EJ, Kang YH, Jang K-S, Lee C, Cho SY (2016). Solution synthesis of telluride-based nano-barbell structures coated with PEDOT:PSS for spray-printed thermoelectric generators. Nanoscale..

[47] Gao L (2019). All paper-based flexible and wearable piezoresistive pressure sensor. ACS Appl. Mater. Interfaces..

[48] Oh J (2019). Highly uniform and low hysteresis piezoresistive pressure sensors based on chemical grafting of polypyrrole on elastomer template with uniform pore size. Small..

[49] Bae GY (2016). Linearly and highly pressure-sensitive electronic skin based on a bioinspired hierarchical structural array. Adv. Mater..

[50] Zhao T (2020). Pollen-shaped hierarchical structure for pressure sensors with high sensitivity in an ultrabroad linear response range. ACS Appl. Mater. Interfaces..

[51] Amjadi M, Kyung K-U, Park I, Sitti M (2016). Stretchable, skin-mountable, and wearable strain sensors and their potential applications: a review. Adv. Funct. Mater..

[52] Cheng W (2018). Flexible pressure sensor with high sensitivity and low hysteresis based on a hierarchically microstructured electrode. IEEE Electron Device Lett.

[53] Wang Z (2019). Full 3D printing of stretchable piezoresistive sensor with hierarchical porosity and multimodulus architecture. Adv. Funct. Mater..

[54] Tang Z, Jia S, Zhou C, Li B (2020). 3D printing of highly sensitive and large-measurement-range flexible pressure sensors with a positive piezoresistive effect. ACS Appl. Mater. Interfaces..

[55] Yan H (2022). 3D printing of dual cross-linked hydrogel for fingerprint-like iontronic pressure sensor. Smart Mater. Struct..

[56] Yin X-Y (2019). 3D printing of ionic conductors for high-sensitivity wearable sensors. Mater. Horizons..

[57] Yin YM (2021). Facile fabrication of flexible pressure sensor with programmable lattice structure. ACS Appl. Mater. Interfaces..

[58] Peng S (2021). Tailored and highly stretchable sensor prepared by crosslinking an enhanced 3D printed UV‐curable sacrificial mold. Adv. Funct. Mater..

