# Supplementary material for: 3D-printing-assisted flexible pressure sensor with a concentric circle pattern and high sensitivity for health monitoring
Source: Microsyst Nanoeng. 2023 Apr 5;9:44. doi: 10.1038/s41378-023-00509-z (PMC10076430; doi:10.1038/s41378-023-00509-z)
Supplement: Supplementary file 1 — Supplementary Information [file 41378_2023_509_MOESM1_ESM.docx]

**Supplementary Information**

**3D-printing-assisted flexible pressure sensor with a concentric circle pattern and high sensitivity for health monitoring**

Jihun Lee^1^, and Hongyun So^1,2^

^1^Department of Mechanical Engineering, Hanyang University, Seoul 04763, South Korea

^2^Institute of Nano Science and Technology, Hanyang University, Seoul 04763, South Korea

Corresponding author: H. So ([hyso@hanyang.ac.kr](mailto:hyso@hanyang.ac.kr))





Fig. S1 Cone modeling in CAD. Schematic of sketching parameters: inner length; inner height; upper thickness; angle. Two inclined lines are parallel. By revolving the sketch, 3D cone modeling was obtained (inset image).





Fig. S2 Verification of unfolded PLA cone using compression method under 2-kg weight. a 3D printed PLA cone with 0.1 mm PLH; b Unfolded PLA cone after compression method with 2-kg weight; c Uniform thickness of 0.31 mm for three positions of compressed PLA cone.





Fig. S3 Optical images of compressed PLA with 0.1 mm PLH under various compressing times. a 5 min; b 2 min; c 50 s.





Fig. S4 Optical images of compressed PLAs each PLH after compression method. a 0.1 mm PLH; b 0.12 mm PLH; c 0.14 mm PLH; d 0.16 mm PLH. (scale bar = 1mm)





Fig. S5 Comparison between direct printing in 2D and compression method for 0.1 mm PLH. Top view of PLA planes fabricated by a1 directly printing in 2D using a GUIDER Ⅱs (FlashForge Co.)-3D printer; a2 a 3DWOX 2X (Shindoh Co.)-3D printer; and using a3 compression method using a GUIDER Ⅱs-3D printer. Top view of PDMS replicated from PLAs b1 directly printed in 2D using a GUIDER Ⅱs-3D printer; b2 a 3DWOX 2X-3D printer; and using b3 compression method. Cross section view of microstructures of PDMS replicated from PLAs directly printed in 2D using c1 GUIDER Ⅱs-3D printer; c2 3DWOX 2X-3D printer; and by PLA created through c3 compression method.





Fig. S6 Theoretical analysis of width and total perimeter of the CCP on the PDMS with respect to the PLH. a Schematic illustration of 3D printed PLA cone, where L, θ, H, and h of inclined line length, angle, height of the cone, and PLH; b Illustration after compressed method shows the width (w) of CCP microstructure. This step matches fabrication process of Fig. 2a4; c Schematic of CCP on the PDMS for obtaining total perimeter.

Figure S6a shows schematic illustration of the side view of 3D printed PLA cone. Theoretical cone was designed by three parameters: inclined line (L), angle (θ), and height (H) of the cone. Because the cone printed by the FDM-type 3D printer, PLH affected roughness of surface. Here, PLH represented h varied from 0.1 to 0.16 mm (Zoomed in Fig. S6a). The inclined line length was determined $\text{L=H/}\sin\text{θ}$ by trigonometric relation. In addition, the number of layers represented n were calculated by $\text{n=H/h}$. As shown in Fig. S6b, after compression method, n layers became length of $\text{L}\cos\text{θ}$. Thus, each length between valley as width could be obtained by

$$\text{w=}\frac{\text{L}\cos\text{θ}}{\text{n}}\text{=}\frac{\frac{\text{H}}{\sin\text{θ}}\cos\text{θ}\text{ }}{\frac{\text{H}}{\text{h}}}\text{=h}\cot\text{θ}$$

In this study, we set angle (θ) of 30°, the width became $\text{w=}\sqrt{3}\text{h}$ only variable with PLH. From the fabrication process of the pressure senor, PDMS was replicated PLA film. Therefore, the CCP on the PDMS was created opposite structures of PLA plane. However, the width still remained $\text{w=}\sqrt{3}\text{h}$. Figure S6c presents the CCP tips on the PDMS and radius of each circle. The circle radius was $\text{r}_{\text{n}}$ and there were n circles. Here, an i^th^ circle radius $\text{r}_{\text{i}}$ can be expressed i multiply by the width w ($\text{r}_{\text{i}}\text{=iw}$), and i^th^ perimeter was $P_{i}=2\pi r_{i}$. Hence, all radius were known, we could calculate total perimeter of CCP on the PDMS as,

$$\text{P}_{\text{total}}\text{=}\sum_{\text{i=1}}^{\text{n}} \text{P}_{\text{i}}\text{=2πw}\sum_{\text{i=1}}^{\text{n}} \text{i}\text{=2πw}\frac{\text{n(n+1)}}{\text{2}}\text{=πwn}\left( \text{n+1} \right)\text{=πh}\cot\text{θ}\frac{\text{H}}{\text{h}}\left( \frac{\text{H}}{\text{h}}\text{+1} \right)\text{=πH}\cot\text{θ}\text{(}\frac{\text{H}}{\text{h}}\text{+1)}$$

The total perimeter equation had three variables of H, θ, and h. However, two variables (H and θ) were set 4.165 mm (Here, we used average height of cone) and 30 °, respectively. Therefore, only variable is the PLH (h) and it determines the total perimeter, and the equation was $\text{P}_{\text{total}}\text{=22.66(}\frac{\text{4.165}}{\text{h}}\text{+1)}$ in mm unit.





Fig. S7 Depth of microstructures on the CCP surface. Cross sectional SEM images of CCP surface with four different PLHs: a 0.1 mm; b 0.12 mm; c 0.14 mm; d 0.16 mm. (Scale bar = 500 µm). e Measurement depth of the CCP microstructures with respect to the PLH.





Fig. S8 Fabrication process of pressure sensor for health monitoring applications. a Pouring the PDMS onto the mold with 0.4-mm thickness and 15-mm width, and covering CCP surface PLA plane with 0.16 mm PLH; b Peeling the PLA and oxygen plasma treatment onto CCP PDMS; c Dispersing PEDOT: PSS using drop-casting with three droplets (80 µL for each droplet), and drying it at 40 ℃ for 2 h; d Wiring and assembling CCP and flat surfaces for finalization.





Fig. S9 Thickness characterization of CCP-based pressure sensor with 0.16 mm PLH for measuring wrist pulse. Each electrode thickness of a 0.4 mm; b 1 mm; c 2 mm (scale bar: 10 mm). d Current responses of wrist pulse for 5 s with respect to the electrode thickness.





Fig. S10 Curved effect to detect wrist pulse. Pressure sensor attached at wrist with a flat position; b curved position. c corresponding current response on the wrist pulse for 5 s.
